# Shenshuaikang Enema, a Chinese Herbal Remedy, Inhibited Hypoxia and Reoxygenation-Induced Apoptosis in Renal Tubular Epithelial Cells by Inhibiting Oxidative Damage-Dependent JNK/Caspase-3 Signaling Pathways Using Network Pharmacology

**DOI:** 10.1155/2020/9457101

**Published:** 2020-11-17

**Authors:** Hongmei Lu, Xinyi Luo, Yuhua He, Bo Qu, Liangbin Zhao, Mingquan Li

**Affiliations:** ^1^Department of Clinical Medicine, Hospital of Chengdu University of Traditional Chinese Medicine, Chengdu, Sichuan, China; ^2^Department of Teaching of the Great Health Management College, Xihua University, Chengdu, Sichuan, China

## Abstract

**Background:**

Acute kidney injury (AKI) is a common clinically critical illness with serious consequences for the patients. Shenshuaikang enema (SE) is a Chinese herbal compound that is used to treat AKI in clinical practice. However, its mechanism of action remains unclear.

**Aim:**

The aim of this study was to investigate the therapeutic effect of SE and explore the molecular mechanisms using network pharmacology and in vitro experiments.

**Materials and Methods:**

The herb-component-target network was constructed based on network pharmacology. The predicted targets and pathways were validated using in vitro experiments. A renal tubular epithelial cell line (HK-2 cells) was exposed to hypoxia and reoxygenation (H/R) using air-tight conditions for five hours and treated with different concentrations of SE (25%, 50%, and 75%) to assess cell viability and apoptosis and determine the optimal experimental dose. Subsequently, H/R-injured HK-2 cells were pretreated with the optimal SE dose and then randomly divided into three groups, the SE, SE-SP600125 (inhibitor of JNK), and SE-NAC (antioxidant) groups. The cell vitality, apoptosis, and death were evaluated using the cell counting kit 8 (CCK8) and carboxyfluorescein succinimidyl ester/propidium iodide (CFSF/PI) staining. The apoptosis-related protein JNK and Caspase-3 were assessed by Western blot. Expression of JNK and Caspase-3 genes was analyzed using real-time quantitative polymerase chain reaction (RT-qPCR).

**Results:**

123 active components and 226 targets were identified from four herbs that composed the herb-compound-target network based on transcriptomics and network pharmacology analyses. The KEGG pathway analyses revealed that the mitochondrial apoptosis pathway was involved in the therapeutic AKI effects of SE. Cell vitality of H/R-induced HK-2 cells was obviously increased when treating them with SE, and the apoptosis was significantly inhibited, especially in the SE (50%) group at 4 and 12 h after modeling. Pretreatment with antioxidant NAC obviously prevented cell death compared to the SE (50%) group, while no obvious reduction of apoptosis was observed in the SP600125 group. JNK expression level was significantly increased in the SE (50%) group compared to the SP600125 (*P* < 0.01) and the NAC group (*P* < 0.05). Caspase-3 was downregulated in the SE (50%) group compared to the SP600125 (*P* < 0.01) and NAC group (*P* < 0.05). Caspase-3 activation in the SP600125 group was higher than that in the NAC group (*P* < 0.05). Moreover, the oxidative damage-dependent JNK/Caspase-3 pathway was identified in the H/R-injured HK-2 cells by inhibiting the JNK activation and oxidative damage.

**Conclusions:**

Our findings suggested that the H/R-triggered apoptosis in HK-2 cells was abrogated by SE by upregulating the oxidative damage-dependent JNK to trigger suppression of Caspase-3.

## 1. Introduction

Acute kidney injury (AKI) is a frequent critical clinical illness with increasing incidence, serious consequences, and unsatisfactory therapeutic options [1]. The survival rate of AKI patients has been lately improved due to the development of renal replacement therapy. However, there are 1.7 million people dying from AKI every year [2]. In addition, a considerable number of surviving AKI patients progress to end-stage renal disease (ESRD), requiring thus long-term hemodialysis treatment and bringing a heavy economic burden to society. Current therapies exhibit limited efficacy [3, 4], underscoring a critical need for the development of novel therapeutic strategies.

Shenshuaikang enema (SE) is a Chinese treatment that uses a mixture composed of Rhubarb, Astragali radix, safflower, and *Salvia miltiorrhiza* in a ratio of 1.5 : 1.5 : 1 : 1, which is administered through the rectum [5]. This administration route avoids influences of the first-pass effect on drug availability seen with oral administration, and it is also convenient for patients. SE has been widely used for more than 50 years in China because of its extensive therapeutic properties with minimal adverse effects. It has been proven by basic experiments that SE can alleviate kidney edema in rabbits and decrease renal tubular degeneration and necrosis in rabbits with AKI [5]. Furthermore, it can promote the repair of necrotic cells in renal tubules. However, the mechanism of action of SE is complex. The effect of SE in AKI and the detailed underlying molecular mechanisms have not yet been clarified.

It has been previously demonstrated that renal tubular epithelial cell necrosis caused by renal ischemia-reperfusion injury (RIRI) is an essential mechanism in AKI, including calcium overload, free radical disorder, endoplasmic reticulum stress, mitochondrial damage, and inflammation [6, 7]. Mitochondria oxidative damage plays an important role in renal tubular epithelial cell apoptosis caused by IRIR. Activators of transcription 3 (Caspase-3), main member of Bcl-2 family, is a hallmark of mitochondrial damage. C-Jun N-terminal kinase (JNKs) is participating in the main intracellular signal transduction pathway activated by RIRI. JNK/Caspase-3 singling pathway has been proven to be related to apoptosis [8]. Such mitochondria-induced apoptosis pathways are mainly mediated by enhanced oxidative damage and subsequently by the activation of JNK and Caspase-3. It has been evidenced that JNK has a regulatory effect on the Bcl-2 family by SP600125 abolished *β*-Ecd-induced Bax downregulation in SH-SY5Y cells and by the promotion of a Bcl-2/Bax ratio [9]. It has been indicated that the continuous activation of JNK has been related to the expression levels of Caspase-3 through the activation of the Bcl-2 family, including Bcl-xL, bax, and bcl-2, Bim [10–12]. Indeed, renal tubular epithelial cell apoptosis, caused by ischemia-reperfusion injury, was also closely related to the JNK signaling pathway, and there was a large amount of JNK activation during renal tubular apoptosis [13]. JNK signaling pathway has been demonstrated to promote cell migration and inhibit cell apoptosis induced by vg suppression by upregulating the activity of Caspase-3 [14]. The exact mechanism which is responsible for the anti-AKI effects of SE still remains uncertain. There are few reports on the mechanism of SE for the treatment of AKI. To the best of our knowledge, only one study has reported that the SE components of rhubarb, *Salvia miltiorrhiza*, *Astragalus membranaceus*, and Safflower (CRSAS) can reduce HK-2 cell apoptosis by inhibiting the expression of CHOP [15].

However, SE has multidrug, multicomponent, and multitarget characteristics, making it difficult to screen possible targets associated with AKI. Network pharmacology is developed on the basis of system biology and computer technology [16]. It systematically observes the intervention and influence of drugs on diseases, prompting the mystery of the synergy of multimolecular drugs [17]. We used a combination of network pharmacology and the concept of “disease-target-component-drug” to identify potential targets for experimental validation. Then, these targets were experimentally processed aiming to lay a foundation for the exploration of combination therapies involving traditional Chinese medicine in the future.

## 2. Materials and Methods

### 2.1. Target Selection

We searched the related targets of SE and AKI screening the core targets of SE in AKI by constructing a protein interaction network. This analysis led to the identification of the key mechanism of SE in AKI by performing functional and pathway enrichment of the core targets. All of these analyses were conducted using network pharmacology. The corresponding targets of these compounds were retrieved from TCMSP databases [18]. Given that oral bioavailability (OB) and drug-likeness (DL) are important indicators to assess the feasibility of using a molecule as a therapeutic drug, we established preset criteria (OB > 30.0 and DL > 0.18) to screen the components of SE [19]. OMIM, TTD, GAD, PharmGkb, and DiGSeE disease-gene databases were mined to discover the targets for AKI. A human protein-protein interaction network was obtained from the Human Protein Reference Database, and Cytoscape 3.7.0 software was used to visualize a protein interaction network. The KEGG pathways were analyzed for enrichment of the predicted targets using the DAVID Database and OmicShare tool database management tools.

### 2.2. Reagents and Antibodies

SE was purchased from Hainan Tianyuan Pharmaceutical Factory (Hainan, China). Anti-Caspase-3 and anti-JNK were purchased from Abcam (Cambridge, USA). Anti-GAPDH was purchased from Bioworld (Minnesota, USA). Carboxyfluorescein diacetate, succinimidyl ester (CFSE), and propidium iodide (PI) were purchased from BBI (Markham, USA). Medium and fetal bovine serum (FBS) were purchased from Gibco (Grand Island, USA). West dura stable peroxide buffer and West dura luminol enhancer solution were purchased from Thermo (Massachusetts, USA).

### 2.3. SE-Containing Serum Preparation

Nine healthy, SPF, New Zealand white rabbits (both male and female), weighing 2000–3000 g were obtained from the Chengdu Dashuo Experimental Animal Co., Ltd. (Chengdu, China) (SCXK2013-14). All experiments were conducted in compliance with the guide for the Care and Use of Laboratory Animals and approved by the Ethics Committee for the Use of Experimental Animals at the Hospital of Chengdu University of Traditional Chinese Medicine. The rabbit population was divided into blank group (conventional feeding), SE group (enema with SE, 9.617 mg/kg/d, according to the conversion formula of human and rabbit body surface area), and PBS group (enema with the same dose of PBS). Enemas were given three times a day for three consecutive days. After the enema in the morning of the fourth day, rabbits were subjected to ear vein anesthesia with 3% pentobarbital sodium (30 mg/kg), the abdominal was opened, and 5 ml blood was collected from the abdominal aorta. These animals were euthanized after the experiment. PBS-containing serum, blank serum, and SE-containing serum samples were obtained by centrifuging, and blank serum was mixed with the SE serum to prepare SE serum of SE (25%), SE (50%), and SE (75%) for use.

### 2.4. Establishment Model of H/R and Grouping

The renal tubular epithelial HK-2 cell line was purchased from Shanghai Aiyan Biotechnology Co., Ltd. (Shanghai, China). The HK-2 cells were cultured in Dulbecco's modified Eagle's medium (DMEM) with 10% fetal bovine serum; then, they were placed in incubators at 37°C with 5% CO_2_ and passaged 1 time every 3–5 days [15]. After being synchronized with culture for 24 h, the HK-2 cells were placed in 24-well plates at a density of 2 × 10^4^–5 × 10^4^ cells per well, and the medium then was covered with tape and sealed for 5 h to simulate the process of ischemia-reperfusion injury. After unsealing, they were randomly divided into 5 groups and added to the blank serum, PBS serum, SE (25%) serum, SE (50%), and SE (75%), observing the apoptosis/death rate and selecting the optimal exposure SE dose. The abovementioned modeling method was repeated adding selected containing serum to the HK-2 cell culture plates after reoxygenation. These treated HK-2 cultures were divided into three H/R model and experimental groups, including SE-treated, SE-SP600125 (a JNK inhibitor)-treated, and SE-NAC (a ROS scavenger)-treated.

### 2.5. Cell Vitality Assay

Cell Counting Kit-8 (CCK-8) was purchased from Dongren Chemical Technologies (Shanghai, China), and it was used to detect the vitality of HK-2 cells. The HK-2 cells were seeded into 96-well plates at a density of 5 × 10^3^ cells per well, adding then 10 *μ*L CCK-8 solution per well at 4 h and 12 h after establishing the H/R model, and incubating at 37°C for 1 h. The absorbance at 450 nm was read with a microplate reader.

### 2.6. Cell Apoptosis Assay

Apoptosis of HK-2 cells was performed with the Certified Functional Safety Expert/Propidium Iodide (CFSE/PI) staining. HK-2 cells were placed at a 24-well plate (2 × 104–5 × 104 cells/well), and CFSE (500 *μ*L–5 *μ*M) was dropped to incubate. The CFSE was then discarded and the cells were washed with PBS. The PBS was removed, 200 *µ*l of PI was added to each well, and the cells were stained at room temperature in the dark for 5 min and then observed using a fluorescence microscope.

### 2.7. Real-Time Quantitative Polymerase Chain Reaction (RT-qPCR)

RT-qPCR was used to measure the mRNA levels for Caspase-3 and JNK. 1 mL Trizol (Sangon Biotech) was added to lyse the cells, which were centrifuged at 12,000 g and 4°C for 5 min. 400 *μ*l of the upper water phase was placed into a clean centrifuge tube, 80 *μ*l chloroform was added (volume ratio CHCl3 : Trizol = 1 : 5), and then cells were centrifuged again at 12,000 g and 4°C for 10 min. The supernatant was discarded, and 1 mL 75% ethanol was added to cause precipitation, centrifuged again at 7,500 g and 4°C for 20 min, and dried at room temperature for 5 min. 30 *μ*l of nuclease-free water was used to lytic RNA precipitation. Then, reagents were added in sequence, as presented in Table 1, to mix with 9 *μ*L of the system solution. The sample was incubated at temperature at 65°C–70°C for about 5 min and then incubated in an icebox for about 2 min. Subsequently, the sample was diluted 20–50 times and was saved at −20°C. RT-qPCR was then performed by an MX3000P Real-Time Fluorescence Quantitative PCR System (Strata-gene, USA) using the following protocol: denaturation at 95°C for 3 min and then 40 cycles of 95°C for 12 s and 62°C for 40 s. Glyceraldehyde 3-phosphate dehydrogenase (GAPDH) was used as an internal control. Primers for the amplification of target genes were designed, and then pairs of primers were selected using the BLAST tool in the PubMed database.

### 2.8. Western Blotting

Analysis with Western blots was conducted to investigate proteins related to JNK and Caspase-3. Cell Total Protein Extraction Kit (Sangon Biotech) was used to extract total cell protein, and 10% sodium dodecyl sulfate-polyacrylamide gel electrophoresis (SDS-PAGE) was used to separate 20 mL of total protein per well. After electrophoresis, the separated proteins were transferred to a polyvinylidene fluoride (PVDF) membrane which was soaked in methanol for 2 min and then cleaned once with the membrane transfer liquid. At 4°C, the regulating voltage was set to 90 V and was kept running for 1 h–1.5 h following one and two fight incubations. Finally, the membranes were exposed to X-ray films, and the resultant films were photographed with the X-Omat BT Film (Kodak, NY, USA). Protein quantification was then performed with a Gel-Pro Analyzer 4.0 (Media Cybernetics, WDC, USA), and bands with a positive response were quantified by densitometry and normalized against GAPDH.

### 2.9. Statistical Analysis

The data were expressed as means ± standard deviation. A Student's *t*-test was used for all pairwise comparisons, and a *p* value threshold of 0.05 was used to infer statistically significant changes. Statistical Product and Service Solutions 21.0 (Software, USA) was used for all other data analyses.

## 3. Results

### 3.1. Analysis of SE Active Compounds and Potential Targets

**Figure 1:**
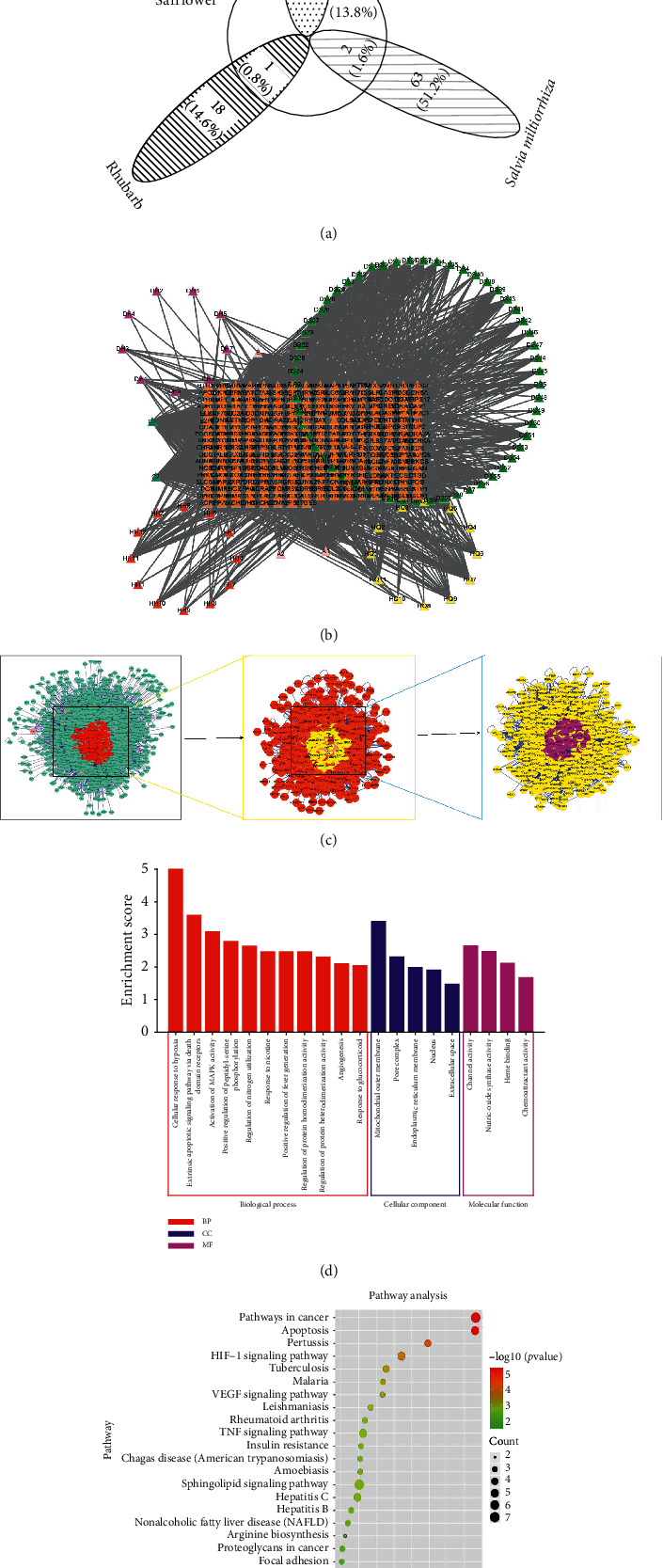
(a) The target for SE, obtained from Rhubarb, *Salvia miltiorrhiza*, Safflower, and Astragalus radix. (b) The network of targets and components of SE. Violet, yellow, green, and red points, respectively, represent Rhubarb, Astragalus radix, *Salvia miltiorrhiza*, and Safflower active components. The orange points represent targets. The lines represent interaction. (c) The flow of searching core targets shared by CRSAS and AKI. First, 1,444 targets were searched. Second, with reference to the double median value of 58 degrees, 386 core targets were selected. Then, 206 core targets were obtained with reference to the median 4745.07 and 0.543467 of the network parameters, betweenness, and closeness to the center, respectively. (d) Enrichment analysis of GO biological process of SE active ingredient potential therapeutic target for AKI using biological process (BP), cellular component (CC), and molecular function (MF) terms. The red, blue, and purple represent BP, CC, and MF, respectively. (e) Pathway enrichment analysis of the core targets of SE (Rhubarb, Astragalus radix, *Salvia miltiorrhiza*, and Safflower), as shown in the bubble chart. The green-red gradient color map represents low to high enrichment and the black circle represents the count.

The results showed that SE contains a total of 123 effective chemical components, and 163 targets were obtained from *Salvia miltiorrhiza*, Safflower, Astragalus radix, and Rhubarb. The components are depicted in Figure 1(a). The composition-target network diagram showed that each component of SE has multiple targets, and one target can be targeted by multiple components (Figure 1(b)). These active components can be the target of SE in AKI.

40 targets in AKI were mined from OMIM, TTD, GAD, etc., and 163 targets in SE were used to construct a PPI network. There were 1444 targets analyzed from AKI and SE. The following network centralities were used to screen the core targets: degree (58), betweenness (4745.07), and closeness centrality (0.543467). 206 core targets were finally screened.

The 206 important genes were analyzed using OmicShare tools to find the related pathways and targets shown in Figure 1(d) and F.

The GO functional enrichment analysis using biological process (BP), cellular component (CC), and molecular function (MF) showed that the predicted targets were enriched to hypoxia, mitochondrial outer membrane, and channel activity (Figure 1(d)). Those biological processes may represent one of the SE mechanisms for exerting AKI treatment effects.

The KEGG enrichment analysis revealed that the targets were enriched to the pathway in cancer, apoptosis, pertussis, HIF-1 singling pathway, and some cancer-related pathways (Figure 1(e)). When considering these results in combination with the GO enrichment analysis results, it is easily inferred that apoptosis induced by mitochondrial damage might be highly related to SE components that are active in the treatment of AKI. This assumption was validated in subsequent experiments of the present study.

### 3.2. SE Inhibited the Apoptosis of HK-2 Cells Induced by H/R

**Figure 2:**
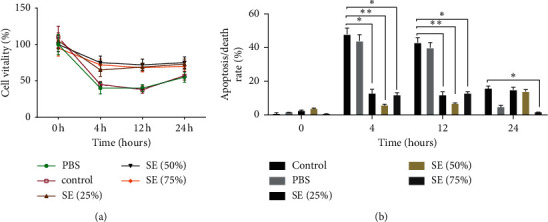
Effect of SE on cell viability and cell death on HK-2 cells induced by H/R. The viability was checked by Cell Counting Kit-8 (CCK-8); cell death was analyzed by Annexin V and propidium iodide (PI) with staining being followed by flow cytometry; ^∗^*P* < 0.05, ^∗∗^*P* < 0.01 versus the control group. SE (Rhubarb, Astragalus radix, *Salvia miltiorrhiza*, and Safflower).

SE serum presented higher efficacy in increasing cell vitality compared with control and PBS as depicted in Figure 2(a). Cell death was analyzed to determine whether the protection of SE as observed above is apoptosis-related. Cell death assay showed that a large number of apoptotic cells were detected in the control and PBS groups, while the apoptosis in the SE groups was significantly lower than the one of the control and PBS groups (*P* < 0.05, *P* < 0.01) from 4 h to 12 h, especially in the SE (50%) group. At 24 h after modeling, there were no obvious differences in cell vitality and apoptosis between groups. These results indicate that SE-containing serum predominantly increased cell vitality and inhibited cell apoptotic death.

### 3.3. SE Inhibited the Apoptosis of HK-2 Cells Injured by H/R via Reducing Oxidative Damage

**Figure 3:**
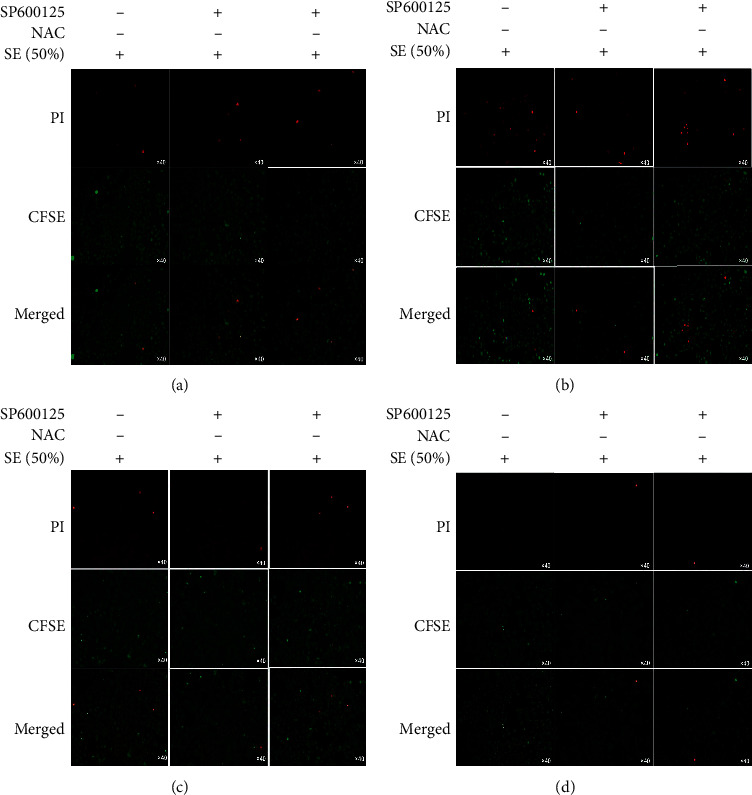
After being treated with SE (50%), H/R-induced HK-2 cells were divided into 3 groups: SE (50%), SE (50%)-NAC group, and SE-SP600125 group. Apoptosis observation was performed at 0 h, 4 h, 8 h, and 12 h after modeling; NAC is a scavenger of oxidative damage and SP600125 is a scavenger of JNK; CFSE/PI was used to observe apoptotic cells. Red bright lights stand for apoptotic cells; SE (Rhubarb, Astragalus radix, *Salvia miltiorrhiza*, and Safflower).

To determine whether inhibition of apoptosis was related to the reduction of mitochondrial oxidative damage, antioxidants NAC and JNK inhibitor SP600125 were used to treat H/R-induced HK-2 cells after SE (50%) pretreatment. Cell apoptosis assay analysis was also performed. Cell apoptosis observation results showed that apoptotic chromatin condensation was clearly decreased in the cells treated with SE (50%) and NAC compared to the SP600125 group at 4 h and 8 h (Figure 3).

This assay revealed a significant decrease in the death of H/R-induced HK-2 cells in response to SE and antioxidants treatment, while blocking JNK activation does not have an obvious effect.

### 3.4. SE Suppressed Apoptosis by Reducing Oxidative Damage-Medicated JNK Activation

**Figure 4:**
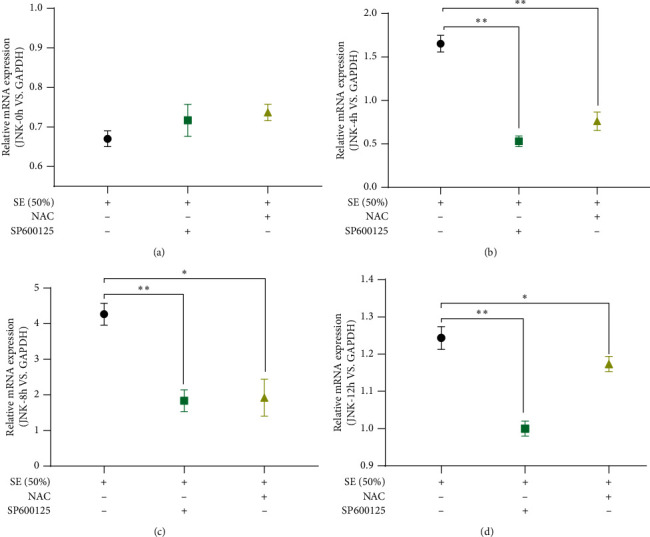
RT-qPCR was used to examine the expression of JNK at 0 h, 4 h, 8 h, and 12 h after modeling; GAPDH was used as an internal reference; *P* < 0.05^∗^, *P* < 0.01^∗∗^. SP600125 is an inhibitor of JNK and NAC was an inhibitor of oxidative damage; SE (Rhubarb, Astragalus radix, *Salvia miltiorrhiza*, and Safflower).

**Figure 5:**
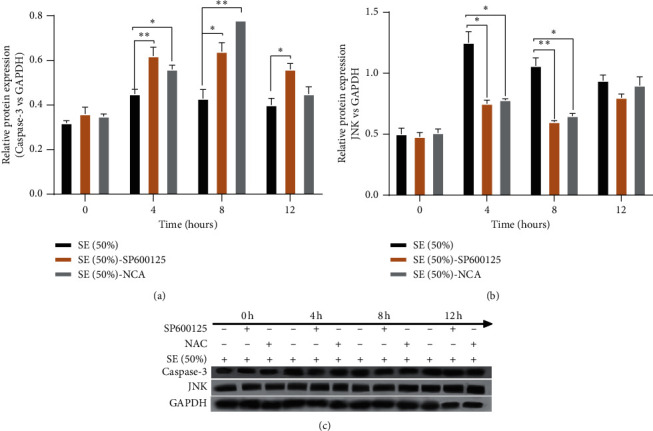
Western blot was used to detect the protein expression of JKN and Caspase-3 at 0 h, 4 h, 8 h, and 12 h after modeling; GAPDH was used as an internal reference; *P* < 0.05^∗^, *P* < 0.01^∗∗^ versus SE (Rhubarb, Astragalus radix, *Salvia miltiorrhiza*, and Safflower).

JNK participates in the mitochondrial apoptosis pathway, forcing the mitochondrial outer membrane permeability transition pores to remain open and the membrane potential decrease. The RT-qPCR data are depicted in Figure 4. At 0 h, the expression of JNK was not statistically different between groups (Figure 4(a)). In contrast to the SP600125 (JNK inhibitor) and NAC (antioxidants), SE was found to have a notably higher potent effect on upregulating JNK at 4 h, 8 h, and 12 h after modeling (*P* < 0.05, *P* < 0.01) (Figures 4(b)–4(d)). When blocking oxidative damage, JNK expression was not significantly differentiated from its value in the SP600125 group (Figures 4(b) and 4(c)). The Western blot results showed that the JNK expression level was clearly decreased in the SE group, compared to the NAC and SP600125 groups (*P* < 0.05, *P* < 0.01) (Figures 5(a) and 5(c)), and no obvious statistical difference between NAC and SP600125 group was observed.

Our data showed that SE suppressed apoptosis mainly by increasing oxidative damage-medicated JNK activation.

### 3.5. SE Inhibited Apoptosis Dependent on Downregulating Oxidative Damage-Medicated JNK/Caspase-3 Signaling Pathway in the H/R-Induced HK-2 Cells

**Figure 6:**
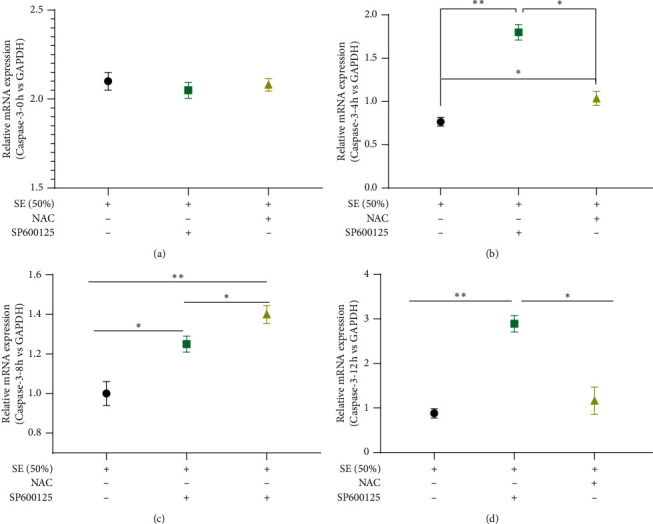
RT-qPCR was used to examine the expression levels of Caspase-3 at 0 h, 4 h, 8 h, and 12 h after modeling; GAPDH was an internal reference; *P* < 0.05^∗^, *P* < 0.01^∗∗^. SP600125 is an inhibitor of JNK, and NAC was an inhibitor of oxidative damage; SE (Rhubarb, Astragalus radix, *Salvia miltiorrhiza*, and Safflower).

Caspase-3, a facilitator of mitochondrial-related apoptosis, is the key execution factor of apoptosis. JNK/Caspase-3 singling pathway plays an important role in mitochondrial oxidative damage-induced apoptosis. Gene expression profiles are presented in Figure 6. At 0 h after modeling, the level of Caspase-3 was obviously diversified (Figure 6(a)). The Caspase-3 mRNA was obviously downregulated in the SE group at 4 h, 8 h, and 12 h after modeling compared to the SP600125 and NAC groups (*P* < 0.05, *P* < 0.01) (Figures 6(b)–6(d)). Surprisingly, when oxidative damage was blocked, the Caspase-3 level was visibly lower compared to its expression profile in the SP600125 group at 4 h, 8 h, and 12 h after modeling (*P* < 0.05) (Figures 6(b)–6(d)). Western blot results showed that the Caspase-3 level was downregulated in the SE group in comparison with the SP600125 and NAC groups (*P* < 0.05) (Figures 5(b) and 5(c)). Furthermore, a noteworthy statistically significant difference between SP600125 and NAC groups was found (Figure 5). These assays showed that SE can inhibit apoptosis by decreasing the expression of Caspase-3. The latter is achieved by upregulating the oxidative damage-medicated JNK activation.

**Figure 7:**
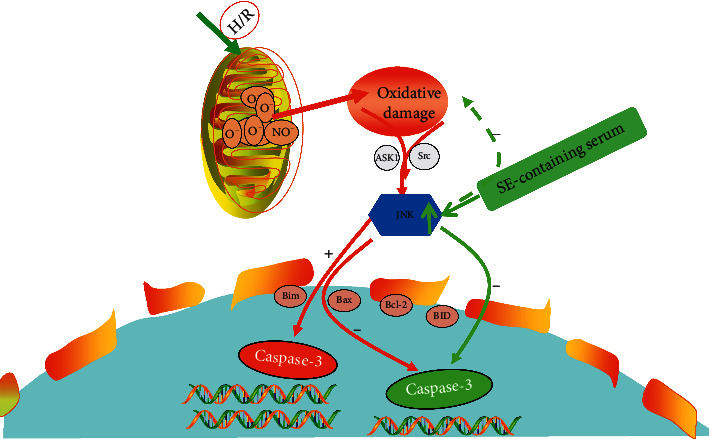
SE inhibited apoptosis of H/R-induced HK-2 cells by upregulating the mitochondrial damage-dependent-JNK expression to downregulate Caspase-3 expression, modulating the JNK/Caspase-3 signaling pathways. H/R: hypoxia/reoxygenation. The solid arrows represent positive feedback; the dotted arrows represent negative feedback. The red line represents the JNK/Caspase-3 signaling pathway; The green represents the action route of SE and target.

The results of the present study indicated that SE suppresses apoptosis of H/R-induced HK-2 cells and this is related to upregulating oxidative damage-medicated JNK activation to trigger downregulation expression of Caspase-3, executing apoptosis (Figure 7). At the same time, SE may participate in other oxidative damage-medicated apoptosis singling pathways, and further studies are required to validate this assumption.

## 4. Discussion

The health and economic burden of AKI is significant. It poses a serious threat to the long-term kidney health of the patients and is a major clinical problem. Mitochondrial damage can activate a variety of apoptosis singling pathways leading to apoptosis of renal tubular epithelial cells and this is the major mechanism of AKI [20]. It has already been reported that mitochondrial oxidative damage in the body can activate JNK/Caspase-3 signaling pathways and is involved in cell survival or antiapoptosis [21, 22]. The molecular mechanism by which JNK suppresses apoptosis is not completely known and understood. It has been proven that JNK-mediated antiapoptosis is involved in the suppression of the prosurvival protein Bcl-xL and the inhibition activation of apoptosis promoter protein Caspase-3 [22, 23]. The activated JNK enters the nucleus and then activates the nontranscription factor Bcl-2 family members (bcl-2, Bim, Bcl-xl, etc.), leading to a decrease of Caspase-3 expression [24]. Evidence has been accumulated that the oxidative damage-medicated JNK/Caspase-3 singling pathway is a potential treatment target for AKI.

SE is a Chinese herbal compound, composed of Rhubarb, *Salvia miltiorrhiza*, Astragali radix, and Safflower, which is mainly used for treating AKI. The clinical application and in-depth development of SE have previously been limited because of the limited existing knowledge of its molecular mechanism. SE has the characteristics of multidrugs, multicomponent, and multitarget, and these traits hinder the research on its specific mechanism. We have initially used network pharmacology to screen the main targets of SE for the treatment of AKI. The KEGG enrichment results of 206 core targets showed that the inhibition of apoptosis induced by the mitochondrial apoptosis pathway was highly related to SE active components for AKI treatment.

Based on network pharmacology, we have used in vitro experiments to observe the apoptosis and mechanism of SE on H/R-injured HK-2 cells. Our results showed that SE-containing serum can indeed promote cell vitality and inhibit the apoptosis of HK-2 cells injured by H/R, and the effect was more significant from 4 h to 12 h after modeling. Unexpectedly, this effect is not dose-dependent, and the practical effect of SE (50%) was evident. The inhibitory effect of time from 4 h to 12 h is obviously considered to be related to the gradual disappearance of the modeling effect. SE (50%) serum was more effective compared to SE (75%), considering the dual effect of active ingredient emodin (ingredients of Rhubarb) on apoptosis. Emodin has been shown to prevent H/R-induced apoptosis, and emodin pretreatment significantly inhibited the phosphorylation of extracellular signal-regulated protein kinase and MAPKs induced by H/R [25]. It has been confirmed that Aloe-Emodin protects against myocardial infarction via the upregulation of miR-133 which is an inhibitor of ROS production and suppressor of the Caspase-3 apoptotic signaling pathway [26]. However, it also has been demonstrated that HK-2 cells are sensitive to emodin-induced cytotoxic effects, which are mediated through the induction of apoptosis in the Caspase-3-dependent pathway [27]. It is possible that different concentrations of emodin may have different effects on different apoptosis pathways, resulting in different effects of renal tubular epithelial cells.

Our data showed that the SE can inhibit the apoptosis of H/R-injured HK-2 cells by inhibiting the oxidative damage-medicated JNK/Caspase-3 singling pathway. Our results showed that SE upregulated expression of survival factor JNK to inhibit activation of downstream Caspase-3. After pretreating with SE, the apoptosis of H/R-induced HK-2 cells was clearly inhibited, the activation of JNK was increased, and the expression of Caspase-3 was decreased in comparison to the SP600125 and NAC groups (*P* < 0.05, *P* < 0.01). When oxidant stress was blocked by NAC, the H/R-induced apoptosis was suppressed, the activated-JNK was lower, and Caspase-3 was higher than the one of the SE-treated group. Apoptosis was observed when the JNK activation was blocked by SP600125, and it was accompanied by downregulation of JNK and upregulation of Caspase-3 in comparison to the SE-treated group (*P* < 0.05). All the data suggested that SE effectively inhibited apoptosis of H/R-induced HK-2 cells by suppressing the expression of Caspase-3. Moreover, this study demonstrated that Caspase-3 was clearly downregulated after SE treatment by upregulating activation of oxidative damage-medicated JNK. Caspase-3 constitutes a central part of the apoptotic machinery and is the hallmark of the mitochondrial apoptosis pathway, a pathway that can be regulated by JNK. JNK participated in the apoptosis process by being involved in mitochondrial oxidative damage [28]. The threat of oxidative damage is particularly significant to DNA, and singling through the JNK pathway is a key cellular response to oxidative damage [29]. JNK signaling can lead to DNA repair, antioxidant production, or cell death depending on the intensity and duration of the damage signal. It has been reported that protection of I/R injury could be achieved through the antiapoptotic effect of the activity of JNK and p38/MAPK pathway, supporting the evidence of the antiapoptosis effect of JNK [30]. In this study, SE was proven to upregulate oxidative damage-dependent JNK activation in H/R-induced HK-2 cells. Activated-JNK triggered the bcl-2 family, reducing the expression of Caspase-3 to promote apoptosis. Therefore, the JNK/Caspase-3 singling pathway may be a key treatment target for AKI. The upregulation of Caspase-3 regulated by JNK results in CAD-mediated DNA fragmentation, chromatin condensation, and membrane blebbing, thereby inducing apoptosis [31]. It has been confirmed that miR-145 inhibited apoptosis of gastric mucosal via upregulating JNK-mediated cytoprotective autophagy. Cell death became potential and activated caspase-3 was upregulated when blocking JNK activation with SP600125 [32]. The assays of the present study showed that SE inhibited apoptosis by upregulating oxidative damage-medicated JNK activation to trigger the suppression of Caspase-3 expression levels.

In summary, SE reduced apoptosis in H/R-injured HK-2 cells via an oxidative damage-dependent JNK/Caspase-3 singling pathway. Unexpectedly, there were statistically significant differences in the expression of Caspase-3 in SE-SP600125 and SE-NAC groups, respectively. Obviously, it does not completely act on the oxidative damage-medicated JNK/Caspase-3 signaling pathways. At the same time, the network pharmacological results suggested that the role of endoplasmic reticulum stress (ERS) is involved in the inhibition of renal tubular epithelial cell apoptosis by SE, reflecting the characteristics of Chinese herbal compound multitargets. This study is an in vitro study, and other predicted signaling pathways by network pharmacology were not detected. Additional in vivo and in vitro experiments should be performed to further validate our findings and hypothesis.

## 5. Conclusion

In conclusion, we evaluated the antiapoptosis efficacy and mechanism of SE by an H/R-induced HK-2 cell model, combining network pharmacology and in vivo experiments. These results revealed that SE can inhibit the apoptosis of H/R-induced HK-2 cells by upregulating oxidative damage-dependent JNK to trigger the suppression level of Caspase-3. Meanwhile, our study introduced a network pharmacology method to predict the targets of the Chinese herbal compound, promoting the modernization of traditional Chinese medicine.

## Figures and Tables

**Table 1:** Sequences of the primers used for qPCR.

Gene name	Primer sequences
JNK	F:5′-TGTGTGGAATCAAGCACCTTC-3′
	R:5′-AGGCGTCATCATAAAACGTTC-3′

Caspase-3	F:5′-CATGTACGTTGCTATCCAGGC-3′
	R:5′-CTCCTTAATGTCACGCACGAT-3′

GAPDH	F:5′-ACCACAGTCCATGCCATCAC-3′
	R:5′-TCCACCACCCTGTTGCTGTA-3′

## Data Availability

The data used to support the findings of this study are included with the articles and available in the Supplementary Materials.

## Supplementary Materials

Supplementary Materials The ingredients of SE (Rhubarb, *Salvia*, Astragalus, and Safflower) are shown in Supplementary 1, and the screening process of targets of SE for AKI is shown in Supplementary 2.
