# Supplementary material for: Shenshuaikang Enema, a Chinese Herbal Remedy, Inhibited Hypoxia and Reoxygenation-Induced Apoptosis in Renal Tubular Epithelial Cells by Inhibiting Oxidative Damage-Dependent JNK/Caspase-3 Signaling Pathways Using Network Pharmacology
Source: Evid Based Complement Alternat Med. 2020 Nov 17;2020:9457101. doi: 10.1155/2020/9457101 (PMC7685836; doi:10.1155/2020/9457101)
Supplement: Supplementary Materials — The ingredients of SE (Rhubarb, Salvia, Astragalus, and Safflower) are shown in Supplementary 1, and the screening process of targets of SE for AKI is shown in Supplementary 2. [file 9457101.f1.pdf]

S1: Ingredients of SE(Safflower, Astragalus radix, Salvia miltiorrhiza, Rhubarb) obtained from TCMSP database.

Herbal	Mol ID	Molecule name	OB(%)	DL
Safflower	MOL001771	poriferast-5-en-3beta-ol	36.91	0.75
	MOL002680	Flavoxanthin	60.41	0.56
	MOL002694	4-[(E)-4-(3,5-dimethoxy-4-oxo-1-cyclohexa-2,5-dienylidene)but-2-enylidene]-2,6-dimethoxycyclohexa-2,5-dien-1-one	48.47	0.36
	MOL002695	lignan	43.32	0.65
	MOL002698	lupeol-palmitate	33.98	0.32
	MOL002706	Phytoene	39.56	0.5
	MOL002707	phytofluene	43.18	0.5
	MOL002710	Pyrethrin II	48.36	0.35
	MOL002712	6-Hydroxykaempferol	62.13	0.27
	MOL002714	baicalein	33.52	0.21
	MOL002717	qt_carthamone	51.03	0.2
	MOL002719	6-Hydroxynaringenin	33.23	0.24
	MOL002721	quercetagetin	45.01	0.31
	MOL002757	7,8-dimethyl-1H-pyrimido[5,6-g]quinoxaline-2,4-dione	45.75	0.19
	MOL002773	beta-carotene	37.18	0.58
	MOL002776	Baicalin	40.12	0.75
	MOL000358	beta-sitosterol	36.91	0.75
	MOL000422	kaempferol	41.88	0.24
	MOL000449	Stigmasterol	43.83	0.76
	MOL000006	luteolin	36.16	0.25
	MOL000953	CLR	37.87	0.68
	MOL000098	quercetin	46.43	0.28
Astragalus radix	MOL000211	Mairin	55.38	0.78
	MOL000239	Jaranol	50.83	0.29
	MOL000296	hederagenin	36.91	0.75
	MOL000033	(3S,8S,9S,10R,13R,14S,17R)-10,13-dimethyl-17-[(2R,5S)-5-propan-2-yloctan-2-yl]-2,3,4,7,8,9,11,12,14,15,16,17-dodecahydro-1H-cyclopenta[a]phenanthren-3-ol	36.23	0.78
	MOL000354	isorhamnetin	49.6	0.31
	MOL000371	3,9-di-O-methylnissolin	53.74	0.48
	MOL000374	5'-hydroxyiso-muronulatol-2',5'-di-O-glucoside	41.72	0.69
	MOL000378	7-O-methylisomucronulatol	74.69	0.3
	MOL000379	9,10-dimethoxypterocarpan-3-O-β-D-glucoside	36.74	0.92
	MOL000380	(6aR,11aR)-9,10-dimethoxy-6a,11a-dihydro-6H-benzofurano[3,2-c]chromen-3-ol	64.26	0.42
	MOL000387	Bifendate	31.1	0.67
	MOL000392	formononetin	69.67	0.21

	MOL000398	isoflavanone	109.99	0.3
	MOL000417	Calycosin	47.75	0.24
	MOL000422	kaempferol	41.88	0.24
	MOL000433	FA	68.96	0.71
	MOL000438	(3R)-3-(2-hydroxy-3,4-dimethoxyphenyl)chroman-7-ol	67.67	0.26
	MOL000439	isomucronulatol-7,2'-di-O-glucosiole	49.28	0.62
	MOL000442	1,7-Dihydroxy-3,9-dimethoxy pterocarpene	39.05	0.48
	MOL000098	quercetin	46.43	0.28
Salvia miltiorrhiza	MOL001601	1,2,5,6-tetrahydrotanshinone	38.75	0.36
	MOL001659	Poriferasterol	43.83	0.76
	MOL001771	poriferast-5-en-3beta-ol	36.91	0.75
	MOL001942	isoimperatorin	45.46	0.23
	MOL002222	sugiol	36.11	0.28
	MOL002651	Dehydrotanshinone II A	43.76	0.4
	MOL002776	Baicalin	40.12	0.75
	MOL000569	digallate	61.85	0.26
	MOL000006	luteolin	36.16	0.25
	MOL006824	α-amyrin	39.51	0.76
	MOL007036	5,6-dihydroxy-7-isopropyl-1,1-dimethyl-2,3-dihydrophenanthren-4-one	33.77	0.29
	MOL007041	2-isopropyl-8-methylphenanthrene-3,4-dione	40.86	0.23
	MOL007045	3α-hydroxytanshinoneⅡa	44.93	0.44
	MOL007048	(E)-3-[2-(3,4-dihydroxyphenyl)-7-hydroxy-benzofuran-4-yl]acrylic acid	48.24	0.31
	MOL007049	4-methylenemiltirone	34.35	0.23
	MOL007050	2-(4-hydroxy-3-methoxyphenyl)-5-(3-hydroxypropyl)-7-methoxy-3-benzofurancarboxaldehyde	62.78	0.4
	MOL007051	6-o-syringyl-8-o-acetyl shanzhiside methyl ester	46.69	0.71
	MOL007058	formyltanshinone	73.44	0.42
	MOL007059	3-beta-Hydroxymethyllenetanshiquinone	32.16	0.41
	MOL007061	Methylenetanshinquinone	37.07	0.36
	MOL007063	przewalskin a	37.11	0.65
	MOL007064	przewalskin b	110.32	0.44
	MOL007068	Przewaquinone B	62.24	0.41
	MOL007069	przewaquinone c	55.74	0.4
	MOL007070	(6S,7R)-6,7-dihydroxy-1,6-dimethyl-8,9-dihydro-7H-naphtho[8,7-g]benzofuran-10,11-dione	41.31	0.45
	MOL007071	przewaquinone f	40.31	0.46
	MOL007077	sclareol	43.67	0.21
	MOL007079	tanshinaldehyde	52.47	0.45
	MOL007081	Danshenol B	57.95	0.56
	MOL007082	Danshenol A	56.97	0.52
	MOL007085	Salvilenone	30.38	0.38
	MOL007088	cryptotanshinone	52.34	0.4

	MOL007093	dan-shexinkum d	38.88	0.55
	MOL007094	danshenspiroketallactone	50.43	0.31
	MOL007098	deoxyneocryptotanshinone	49.4	0.29
	MOL007100	dihydrotanshinlactone	38.68	0.32
	MOL007101	dihydrotanshinone Ⅰ	45.04	0.36
	MOL007105	epidanshenspiroketallactone	68.27	0.31
	MOL007107	C09092	36.07	0.25
	MOL007108	isocryptotanshi-none	54.98	0.39
	MOL007111	Isotanshinone II	49.92	0.4
	MOL007115	manool	45.04	0.2
	MOL007118	microstegiol	39.61	0.28
	MOL007119	miltionone Ⅰ	49.68	0.32
	MOL007120	miltionone Ⅱ	71.03	0.44
	MOL007121	miltipolone	36.56	0.37
	MOL007122	Miltirone	38.76	0.25
	MOL007123	miltirone Ⅱ	44.95	0.24
	MOL007124	neocryptotanshinone ii	39.46	0.23
	MOL007125	neocryptotanshinone	52.49	0.32
	MOL007127	1-methyl-8,9-dihydro-7H-naphtho[5,6-g]benzofuran-6,10,11-trione	34.72	0.37
	MOL007130	prolithospermic acid	64.37	0.31
	MOL007132	(2R)-3-(3,4-dihydroxyphenyl)-2-[(Z)-3-(3,4-dihydroxyphenyl)acryloyl]oxy-propionic acid	109.38	0.35
	MOL007140	(Z)-3-[2-[(E)-2-(3,4-dihydroxyphenyl)vinyl]-3,4-dihydroxy-phenyl]acrylic acid	88.54	0.26
	MOL007141	salvianolic acid g	45.56	0.61
	MOL007142	salvianolic acid j	43.38	0.72
	MOL007143	salvilenone Ⅰ	32.43	0.23
	MOL007145	salviolone	31.72	0.24
	MOL007149	NSC 122421	34.49	0.28
	MOL007150	(6S)-6-hydroxy-1-methyl-6-methylol-8,9-dihydro-7H-naphtho[8,7-g]benzofuran-10,11-quinone	75.39	0.46
	MOL007151	Tanshindiol B	42.67	0.45
	MOL007152	Przewaquinone E	42.85	0.45
	MOL007154	tanshinone iia	49.89	0.4
	MOL007155	(6S)-6-(hydroxymethyl)-1,6-dimethyl-8,9-dihydro-7H-naphtho[8,7-g]benzofuran-10,11-dione	65.26	0.45
	MOL007156	tanshinone Ⅵ	45.64	0.3
Rhubarb	MOL002235	EUPATIN	50.8	0.41
	MOL002251	Mutatochrome	48.64	0.61
	MOL002259	Physciondiglucoside	41.65	0.63
	MOL002260	Procyanidin B-5,3'-O-gallate	31.99	0.32
	MOL002268	rhein	47.07	0.28
	MOL002276	Sennoside E_qt	50.69	0.61
	MOL002280	Torachrysone-8-O-beta-D-(6'-oxayl)-glucoside	43.02	0.74

	MOL002281	Toralactone	46.46	0.24
	MOL002288	Emodin-1-O-beta-D-glucopyranoside	44.81	0.8
	MOL002293	Sennoside D_qt	61.06	0.61
	MOL002297	Daucosterol_qt	35.89	0.7
	MOL002303	palmidin A	32.45	0.65
	MOL000358	beta-sitosterol	36.91	0.75
	MOL000471	aloe-emodin	83.38	0.24
	MOL000554	gallic acid-3-O-(6'-O-galloyl)-glucoside	30.25	0.67
	MOL000096	(-)-catechin	49.68	0.24

S2:The screening process of core targets of SE for AKI. SE(Safflower, Astragalus radix, Salvia miltiorrhiza, Rhubarb).

Targets of SE → 163

NOS2, insr, AR, PTGS2, MMP2, TNF, EGFR, HGF, IL6, MMP1, MAPK1, IFNG, JUN, CDK1, XDH, CDK4, TP53, APP, GSTP1, VEGFA, TOP1, HMOX1, RB1, CASP7, IL2, PTGS1, ESR1, PPARG, CA2, Ptpn1, ESR2, DPP4, MAPK14, GSK3B, HSP90, PIK3CG, PRKACA, PRSS1, PIM1, CCNA2, NCOA2, NOS2, BCL2, ALOX5, ODC1, ACACA, MGAM, PLAU, PON1, CTSD, CCL2, IL1B, SELE, MPO, PLAT, GJA1, VCAM1, THBD, F3, NQO1, PTGER3, SOD, CYP3A4, CYP1A2, COL1A1, EGF, POR, SULT1E1, gyrB, COL3A1, Gstm1, HSPA5, ACPP, AHR, Gstm2, F2, Potassium, SCN5A, F10, NOS3, F7, RXRA, ACHE, ADRB2, TOP2, AKR1B1, GABRA1, MMP3, blaC, MAOB, CHEK1, MAP2, DRD1, CHRM3, CHRM1, GABRA2, CHRM4, PDE3A, HTR2A, GABRA5, ADRA1A, GABRA3, PGR, CHRM2, ADRA1B, CHRNA2, SerT, OPRM1, NR3C1, CHRNA7, camC, AKR1C3, MAPK8, PPP3CA, SLC6A2, CALM, ADRB1, ADRA2A, DAT, NR3C2, LTA4H, MAOA, ADH1C, IGHG1, CTRB1, NCOA1, KCNMA1, EGLN1, CYCS, MMP10, CHRM5, HTR3A, OPRD1, ADRA1D, DRD2, ADRA2C, DRD5, HTR1A, HTR2C, ADRA2B, HTR1B, GABRA6, GABRG3, GABRE, CALCR, EDNRA, FASN, ITGB, ECE1, ADH1B, lysoz, cobT, GRIA2, PYGM, PPARD, RXRB, KDR, MT-ND6, ATP5F1B, PKIA, FLT4 , FLT1, BTK

↓

Targets related AKI → 40

CYBA, ACE, HAVCR1, SLC2A9, PTER, CASR, egl-9, ALB, G6PD, CDKN2A, EDN1, IL6, PTGS2, ANTXRL, ICAM1, CST3, TNF, IGF1, HMOX1, NOS3, HGF, NA, ST8SIA2, AQP2, EPO, VEGFA, NOS2, CD4, AKT1, BMP7, NFE2L2, MET, CASP3, EGF, SLC9A3, BAX, BCL2, FAS, FADD, BID

↓

Screening core targets SE for AKI → 206

TRAF2, CCT7, RB1, RASA1, ACTG1, ACTA1, ACTB, RELA, KHDRBS1, PARP1, PLK1, ADRB2, RPL35, CASP8, CASP7, CBL, OBSL1, SYK, PSMA5, PSMA2, PSMA3, PSMD2, PSMC6, PSMC2, MAP3K7, PSMD1, PSMD11, PTBP1, CD4, FN1, TNF, PAN2, PCBP1, PARK2, PCNA, RBX1, TUBB4B, RACK1, NF2, NFE2L2, SYNCRIP, NOTCH1, NOS2, HUWE1, ITCH, TUBB, YBX1, CCDC8, ARRB1, ARRB2, PRKCA, PRKACA, PRKCZ,ATM, PRKCD, PRKCB ,CSNK2A3 ,PRKDC, UCHL5, BAX, CCND1, BARD1, BCL2L1, BCL2, CHUK, CLTC, PIN1, RAF1, IAP, DDX3X, CDC42, CDC5L, DDB1, FBXW7, CDK2, PAXIP1, DDX5, CDH1, CDKN2A, CDKN1A, FBXO6, CDK4, TNF, ERBB2, TOP1, PTPN11, ESR1,TRAF2, EWSR1, PXN, EZH2, TRAF1, TRAF6, HLA-B, RNF2, MAPK14, CSNK2B, CSNK2A2, ABCE1, CSNK2A1, EMD, TUBA1A, RPA2, RPL13, RPL8, RPL7, RPL18, RPL12, RPL11, MTOR, SNCA, GRB2, EED, AR, GSK3B, SP1, FADD, FAS, STIP1, CCT3,YWHAQ, HTT, HDAC2, FBXO25, TUBA4A, RPS4X, PABPC1, RPS14, EZR, VIM, RPS10, RPS12, RPS15, RPS17, RPS28, SKP1, SHC1, FAF2, SKI, SFN, EEF2, KAT2B, EEF1G, HDAC3, HNRNPU, TGM2, HNRNPA2B1, HNRNPD, HNRNPH1, FBXW11, SMARCA4, MAP3K14, CALM3, HIST1H3F, CALM2, BTRC, CALM1, SMN1, LRRK2, YWHAG, XPO1, KRT18, RPSA, NCOA3, LGALS3BP, LCK, LDHA, DNAJA1, HSP90AB1, HSPD1, HSPA9, IKBKB, HIST3H3, HIST1H3B, NCL, MAPT, NEDD4, MCC, MDM2, RELA, TNF, XIAP, RELA, TP53, NFKBIA, FADD, BCL2L1, ATM, AKT1, TNFRSF1A, CASP3, CASP7, BCL2, RIPK1, BAX, NTRK1, CASP8, IKBKG, FAS, IKBKB, MAP3K14, CHUK, PIK3R1, PIK3R

↓

targets related apoptosis → 20

TRAF2, TNF, XIAP, RELA, TP53, NFKBIA, FADD, ATM, AKT1, TNFRSF1A,CASP7, BCL2, BAX, NTRK1, CASP8, IKBKG, FAS, MAP3K14, CHUK, PIK3R
